# Proinsulin Shares a Motif with Interleukin-1α (IL-1α) and Induces Inflammatory Cytokine via Interleukin-1 Receptor 1*

**DOI:** 10.1074/jbc.M116.731026

**Published:** 2016-05-16

**Authors:** Siyoung Lee, Eunsom Kim, Hyunjhung Jhun, Jaewoo Hong, Areum Kwak, Seunghyun Jo, Suyoung Bae, Jongho Lee, Busun Kim, Jungmin Lee, Sulah Youn, Somi Kim, Miyeon Kim, Hyunwoo Kim, Youngmin Lee, Dong-Ki Choi, Yong-Sung Kim, Soohyun Kim

**Affiliations:** From the ‡ Laboratory of Cytokine Immunology, Department of Biomedical Science and Technology and; § College of Veterinary Medicine, Konkuk University, 120 Neungdong-ro, Gwangjin-gu, Seoul 05029, Korea,; ¶ Division of Nephrology, Department of internal medicine, Jeju National University, 63243 Jeju-si, Jeju-do, Korea,; ‖ Department of Medicine, Pusan Paik Hospital, Collage of Medicine, Inje University, Busan 47392, Korea, and; ** Department of Molecular Science and Technology, Ajou University, Suwon 16499, Korea

**Keywords:** cytokine, insulin, interleukin 1 (IL-1), protein motif, toll/interleukin-1 receptor (TIR)*

## Abstract

Although it has been established that diabetes increases susceptibility to infections, the role of insulin (INS) in the immune response is unknown. Here, we investigated the immunological function of INS. Proinsulin dimer (pINSd) was a potent immune stimulus that induced inflammatory cytokines, but mature INS was unable to induce an immune response. An affinity-purified rabbit polyclonal antibody raised against mature IL-1α recognized IL-1α and pINS but failed to detect mature INS and IL-1β. Analysis of the pINS sequence revealed the existence of an INS/IL-1α motif in the C-peptide of pINS. Surprisingly, the INS/IL-1α motif was recognized by monoclonal antibody raised against IL-1α. Deleting the INS/IL-1α motif in pINSd and IL-1α changed their activities. To investigate the pINSd receptor, the reconstitution of IL-1 receptor 1 (IL-1R1) in Wish cells restored pINSd activity that was reversed by an IL-1R antagonist. These data suggested that pINSd needs IL-1R1 for inflammatory cytokine induction. Mouse embryo fibroblast cells of IL-1R1-deficient mice further confirmed that pINSd promotes immune responses through IL-1R1.

## Introduction

Insulin (INS)^3^ is a hormone secreted from particular β-cells of the pancreas that regulates carbohydrate and fat metabolism in the body by triggering cells to absorb glucose. A dog pancreatic hormone has been shown to be essential for metabolizing carbohydrates and the successful treatment of diabetes (1, 2). Human recombinant polypeptide A and B chains of mature INS or pro-INS (pINS) were synthesized in *Escherichia coli* (3) as a result of cloning rat and human INS complementary DNA (4, 5).

Among the 11 cytokines in the interleukin (IL)-1 family cytokine, except IL-1 receptor antagonist (IL-1Ra), 10 have no signal peptide. The signal peptide in IL-1Ra allows the cell to release IL-1Ra as an active molecule without processing (6). IL-1α and IL-1β are essential members of the IL-1 cytokine family, having important roles as immune sentinels early in infections. However, IL-1Ra is a natural antagonist to block IL-1α and IL-1β activity by its competition for binding to IL-1 receptor 1 (IL-1R1) and suppresses the immune response (7).

Nine members of the IL-1R family, except IL-1R8, have three immunoglobulin-like domains in the extracellular domain. The IL-1R family has crucial roles in innate and acquired immune responses against infections because IL-1R family shares a conserved functional domain with Toll-like receptor. The intracellular domain of the IL-1R family contains a Toll/interleukin-1 receptor homology domain that interacts with adaptor molecules for downstream signal pathways, whereas IL-1R2 is a decoy receptor due to the lack of the Toll/interleukin-1 receptor domain in its short intracellular domain (8). It has been established that IL-1α and IL-1β use identical receptor components, IL-1R1 and IL-1R3, to transmit signaling (9, 10), and they induce an immune response against infections.

A decreased proliferative response to different stimuli has been observed in the lymphocytes of diabetic patients in comparison with that of non-diabetic normal control individuals (11). Also an unusual cell-mediated immunity has been described in type 1 and type 2 diabetes patients (11–14). For instance, the different types of primary immune cells from diabetic patients have been studied in the presence or absence of stimuli. Without stimulation, the levels of TNFα in type 1 diabetics, IL-6 in type 2 diabetics, and IL-8 in both types of diabetics were increased compared with nondiabetic controls (15–17). IL-1α/β and IL-6 secretion from PBMCs and monocytes in the presence of LPS stimulation was reduced in both types of diabetics (18), but there were no differences in TNFα concentrations after stimulation with LPS when comparing monocytes of type 2 diabetics with nondiabetic controls (19). The increased cytokines in diabetics could be explained by advanced glycation end products. Various studies have suggested that binding of advanced glycation end products to nondiabetic cells in the absence of stimulation leads to increased cytokine production (20–22).

To date, INS therapy has been solely focused on reducing blood glucose levels, although increasing evidence suggests that INS is important in the immune response following pathogenic infections (23–25) as well as during recovery after surgery (26, 27). The present study provides insight into hitherto unknown immunological functions of proinsulin dimer (pINSd): its ability to induce immune responses through IL-1R1.

## Results

### 

#### 

##### pINSd Induces Inflammatory Cytokines

**FIGURE 1.**
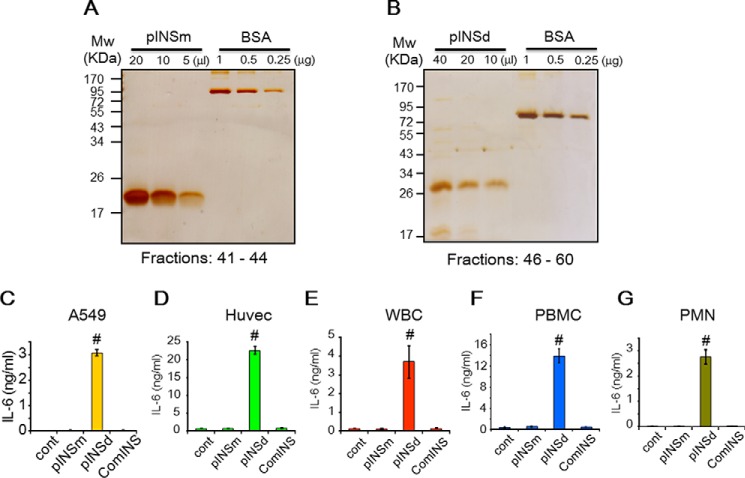
**Expression and bioassay of recombinant pINS.** Recombinant pINSm (*A*) and pINSd (*B*) proteins were pooled from HPLC fractions as indicated at the *bottom* and visualized by silver staining. Biological activity of pINSm, pINSd, and comINS was examined with A549 cells (*C*), Huvecs (*D*), primary human WBCs (*E*), PBMCs (*F*), and PMN leukocytes (*G*). Data in *C–G* are comparisons between the control (*cont*) and pINSd treatment. Data are mean ± S.E. (*error bars*). #, *p* < 0.001 from three replicates.

We expressed recombinant INS to investigate its role in the immune response. Two distinct molecular sizes of proinsulin monomer (pINSm) and pINSd were observed by silver staining (not shown). We used each fraction to stimulate human umbilical vein endothelial cells (Huvecs). Prominent induction of IL-6 was observed where pINSd fractions appeared (not shown). Therefore, the pINSm and pINSd fractions were pooled and confirmed the purity by silver staining as shown in Fig. 1, *A* and *B*. The pINSm and pINSd including a mature commercial INS (comINS) were used to stimulate various cell types. The pINSd was highly effective in inducing IL-6, whereas the pINSm and comINS remained ineffective (Fig. 1, *C–G*). Huvecs and PBMCs were shown to have a very high response to pINSd compared with other cell types. The pINSd and IL-1α stimulation also induced IL-1β and tumor necrosis factor (TNF) α in the primary cells but not in A549 and Huvecs (not shown). Because IL-6 production was most reliable across different immune and non-immune cell types, we chose to monitor IL-6 regulation by pINSd.

##### Identifying a Motif of INS/IL-1α

**FIGURE 2.**
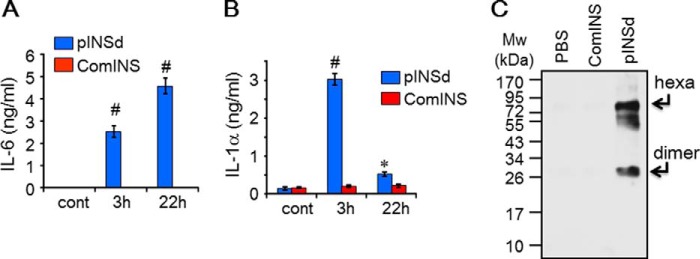
**Detection of a common motif in both IL-1α and pINS.** IL-6 levels in the supernatant of A549 cells treated with pINSd were increased by prolonged incubation (*A*), but IL-1α levels were decreased with prolonged incubation (*B*) as measured by the IL-1α ELISA kit described under “Experimental Procedures.” Incubation time is indicated on the *x axis. C*, Western blot with affinity-purified rabbit polyclonal antibody raised against IL-1α detected 26 (dimer)- and 75 (hexamer (*hexa*))-kDa size bands where pINSd was added but not where PBS or comINS was added. Data in *A* and *B* are comparisons between pINSd treatment and untreated control (*cont*). Data are mean ± S.E. (*error bars*). *, *p* < 0.05; #, *p* < 0.001 from duplicates.

**FIGURE 3.**
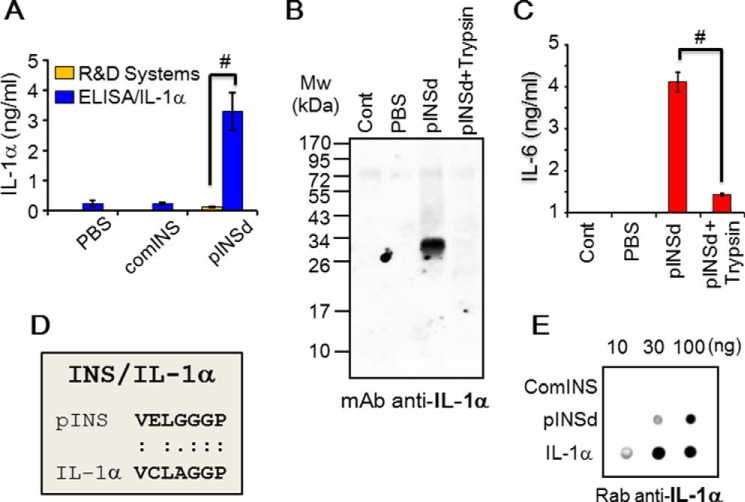
**Identification of INS/IL-1α motif.** *A*, anti-IL-1α mAb ELISA detected pINSd but failed to recognize comINS, which is mature INS lacking the C-peptide. A commercial IL-1α ELISA kit did not recognize pINSd. *B*, pINSd was incubated for 60 min in the presence or absence of trypsin (Sigma-Aldrich). pINSd (100 ng/lane loaded) was properly detected as a band of ∼30 kDa, but pINSd was not observed where trypsin was added. *C*, the same batch of pINSd in the presence or absence of trypsin was examined for IL-6 induction in A549 cells. The production of IL-6 was found to be due to pINSd because trypsin abolished IL-6 production. *D*, sequence analysis of pINS and IL-1α revealed the INS/IL-1α motif. *E*, dot blot of recombinant pINSd and IL-1α protein with the mAb anti-IL-1α. Data in *A* are comparisons between a commercial IL-1α ELISA and IL-1α developed with the mAb anti-IL-1α. Data in *C* are comparisons between the presence and absence of trypsin. Data are mean ± S.E. (*error bars*). #, *p* < 0.001 from three replicates. *Cont*, control.

The pINSd-mediated inflammatory cytokine production in various cell types precisely overlapped with IL-1α activity but not with IL-1β activity (not shown). For instance, although activity levels varied across different cell types, pINSd and IL-1α were active in all cell types. Next, we were interested in investigating how the activity of pINSd in different cells corresponded with IL-1α activity but not with IL-1β. Intriguingly, IL-6 production by pINSd in A549 cells increased in a time-dependent manner (Fig. 2*A*), whereas IL-1α levels decreased with prolonged incubation time (Fig. 2*B*). We used an IL-1α ELISA to detect IL-1α levels in the A549 cell culture supernatant. The IL-1α ELISA kit was developed with a monoclonal antibody (mAb) against mature IL-1α as a capture antibody and an affinity-purified rabbit polyclonal antibody against the same antigen as a detection antibody (see “Experimental Procedures”). We then performed Western blotting with the supernatant of pINSd-treated A549 cells by using the affinity-purified rabbit polyclonal antibody. The polyclonal antibody detected the bands of the dimer and hexamer forms in the pINSd-treated A549 cells but not in those cells treated with comINS (Fig. 2*C*). We compared the results of our IL-1α ELISA with the results from a commercial IL-1α ELISA kit. Surprisingly, our IL-1α ELISA detected pINSd, whereas the commercial kit failed to detect pINSd (Fig. 3*A*). An additional experiment in which trypsin was used to degrade pINSd prior to immunoblotting (Fig. 3*B*) suggested that the added recombinant pINSd is responsible for IL-6 induction in the A549 cell assay because trypsin dramatically abolished its activity (Fig. 3*C*).

**FIGURE 4.**
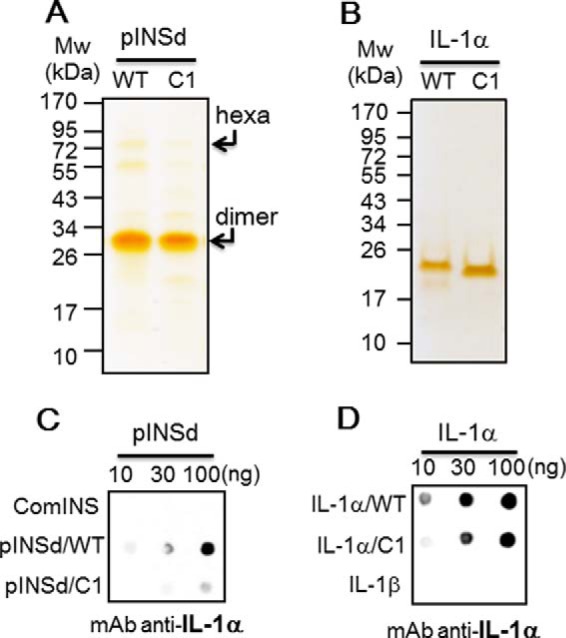
**Deletion of INS/IL-1α motif.** *A*, deletion of motif C1 (7 amino acids) in the C-peptide and the recombinant pINSd/WT and /C1 were visualized by silver staining. The molecular sizes of the hexamer (*hexa*) and dimer pINS forms are indicated by *arrows. B*, deletion of the motif IL-1α/C1 (7 amino acids) at the C terminus. Recombinant IL-1α/WT and /C1 were visualized by silver staining. *C*, dot blot showing that the detection sensitivity by mAb anti-IL-1α was reduced in pINSd/C1 when compared with pINSd/WT. *D*, dot blot showing the sensitivity of anti-IL-1α for IL-1α/C1 compared with IL-1α/WT. The concentration is given at the *top*.

These results illustrate that IL-1α likely shares an epitope with pINS that is recognized by the rabbit polyclonal antibody raised against IL-1α but not with mature comINS (Fig. 3*A*). A careful sequence analysis of pINS and IL-1α revealed an INS/IL-1α motif of 7 amino acid residues, VELGGGP, with 71.4% identity in the C-peptide of pINS (Fig. 3*D*). Alternatively, a dot blotting experiment demonstrated that the mAb anti-IL-1α recognized pINSd but failed to recognize mature comINS (Fig. 3*E*) because the INS/IL-1α motif in C-peptide does not exist in the mature comINS. The rabbit polyclonal antibody has preferential reactivity with hexamer rather than dimer because detection sensitivity of the faint hexamer band was similar to the thick dimer band (Fig. 4*A*). Mature INS is composed of two polypeptide chains, β chain and α chain, but lacks the C-peptide.

##### Deletion of INS/IL-1α Motif in pINSd and IL-1α

To verify whether the INS/IL-1α motif is an anti-IL-1α mAb epitope in the C-peptide, VELGGGP in pINS was deleted using mutagenesis (28). Recombinant pINSd wild type (WT) and pINSd/C1 were expressed and purified (Fig. 4*A*). We examined whether mAb anti-IL-1α recognizes the INS/IL-1α motif by using the pINSd/C1 mutant. As expected, mAb anti-IL-1α recognized the INS/IL-1α motif in the C-peptide of pINSd, and the motif was found to be a critical epitope for mAb anti-IL-1α (Fig. 4*C*).

The INS/IL-1α motif at the C terminus of IL-1α (not shown) was also deleted in the same manner as the motif in pINSd/C1 was deleted. Recombinant IL-1α/WT and /C1 were expressed and purified by high performance liquid chromatography (HPLC) as shown in Fig. 4*B*. IL-1α/WT and /C1 were checked with the mAb raised against IL-1α. Consistently, the sensitivity of detection of IL-1α/C1 by the mAb anti-IL-1α was weak compared with IL-1α/WT (Fig. 4*D*). The mAb anti-IL-1α failed to recognize IL-1β as shown in the *bottom row* of Fig. 4*D* because IL-1β does not possess the motif (not shown).

##### Activity of INS/IL-1α Motif Mutants Varied between pINSd and IL-1α

**FIGURE 5.**
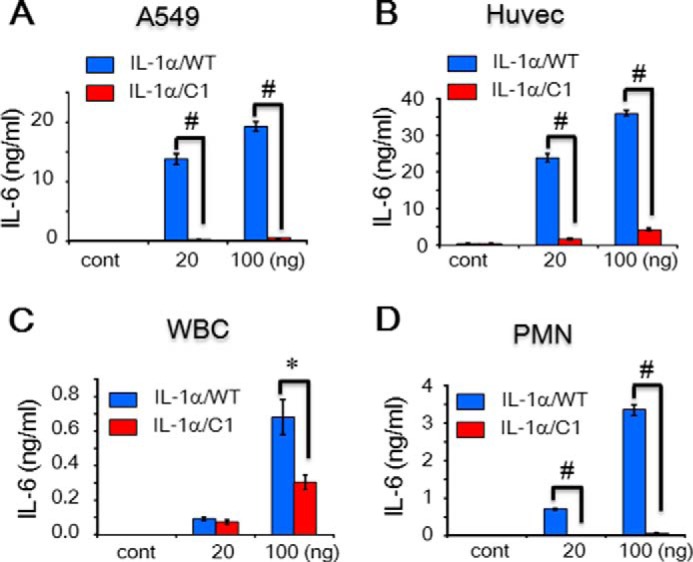
**Bioassay of the motif-deleted IL-1α mutant.** *A–D*, assay of IL-1α/WT and /C1. A549 cells (*A*), Huvecs (*B*), WBCs (*C*), and PMN leukocytes (*D*) were treated with IL-1α/WT and /C1. The INS/IL-1α motif-deleted IL-1α/C1 mutant showed severe loss of activity compared with IL-1α/WT. Concentrations are indicated at the *bottom* of each graph. Data in *A–D* are comparisons between IL-1α/WT and /C1 mutant. Data are mean ± S.E. (*error bars*). *, *p* < 0.05; #, *p* < 0.001 from duplicates. *cont*, control.

**FIGURE 6.**
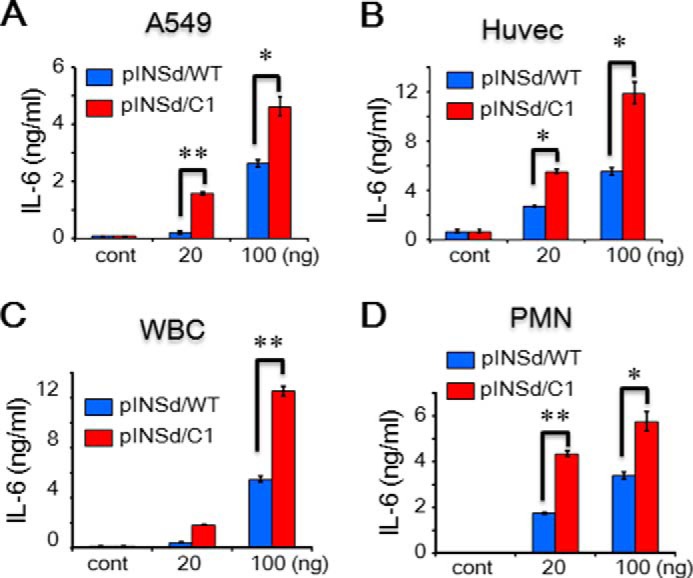
**Bioassay of the motif-deleted pINSd mutant.** *A–D*, assay of pINSd/WT and /C1. A549 cells (*A*), Huvecs (*B*), WBCs (*C*), and PMN leukocytes (*D*) were treated with pINSd/WT and /C1. The INS/IL-1α motif-deleted pINSd/C1 mutant was more active than pINSd/WT across different cell type. Data in *A–D* are comparisons between pINSd/WT and /C1 mutant. Data are mean ± S.E. (*error bars*). *, *p* < 0.05; **, *p* < 0.01 from duplicates. *cont*, control.

Recombinant IL-1α/WT and /C1 were used to stimulate different cell types to examine their activities. IL-1α/C1 completely lost its activity in A549 cells, Huvecs, and polymorphonuclear (PMN) leukocytes (Fig. 5*A*, *B*, and *D*) but was partially impaired in whole blood cells (WBCs) (Fig. 5*C*). Interestingly, pINSd/C1 was more effective at enhancing activity compared with pINSd/WT across cell types (Fig. 6, *A–D*).

##### The Immunological Activity of pINSd via IL-1R1

**FIGURE 7.**
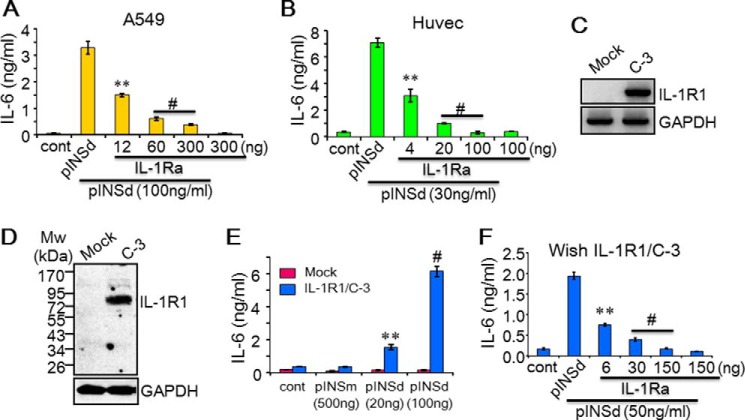
**pINSd-mediated IL-6 production via IL-1R1.** pINSd-induced IL-6 in A549 cells (*A*) and Huvecs (*B*) was effectively suppressed with low concentrations of IL-1Ra. *C–F*, reconstitution of IL-1R1 and IL-1Ra activity in Wish IL-1R1/C-3. IL-1R1 was not detected in the mock control, but IL-1R1 expression was shown in C-3 by RT-PCR (*C*) and Western blotting (*D*). *E*, Wish IL-1R1/C-3 was tested with pINSd including a high concentration of pINSm as indicated on the *x axis. F*, pINSd-mediated IL-6 in Wish IL-1R1/C-3 was specifically inhibited by IL-1Ra. Concentrations of IL-1Ra and pINSd are given at the *bottom*. Data in *E* are a comparison between Wish IL-1R1/C3 and mock control; data in *A*, *B*, and *F* are comparisons between standalone pINSd-treated and IL-1Ra-pretreated cells; concentrations are indicated at the *bottom*. Data are mean ± S.E. (*error bars*). **, *p* < 0.01; #, *p* < 0.001 from three replicates. *cont*, control.

We were interested in testing whether IL-1Ra competes with pINSd because its activity in different cells corresponded with IL-1α. IL-1Ra is a natural receptor antagonist that binds to IL-1R1 and competes with IL-1α and IL-1β (7). pINSd-induced IL-6 was completely inhibited by IL-1Ra in A549 cells and Huvecs in a dose-dependent manner (Fig. 7, *A* and *B*).

Next, we reconstituted IL-1R1 in Wish cells to prove the role of IL-1R1 directly in pINSd activity. Our results revealed that Wish cells do not respond to IL-1 because of lacking IL-1R1 expression.^4^ We first stably expressed IL-1R1 and then examined whether Wish/IL-1R1 clones 3 and 18 (C-3 and C-18) restored IL-1a and IL-1b activity by inducing IL-6 product. IL-1α and IL-1β enhanced IL-6 upon reconstitution of IL-1R1, but the mock control remained inactive (not shown). IL-6 was significantly increased in Wish IL-1R1/C-3 and /C-18 after pINSd stimulation, similar to the enhancement of IL-1α and IL-1β activity, but the mock control did not respond to pINSd (not shown). In addition, IL-1R1 expression was not detected in the mock control; however, IL-1R1 expression in Wish/C-3 was confirmed by RT-PCR (Fig. 7*C*), immunoblotting (Fig. 7*D*), and FACS analysis (not shown). The dose-dependent pINSd-mediated IL-6 production (Fig. 7*E*) was reversed by IL-1Ra (Fig. 7*F*) in Wish/IL-1R1/C-3.

##### Specific Activity of pINSd with Mouse Embryo Fibroblasts (MEFs) of IL-1R-deficient Mice

**FIGURE 8.**
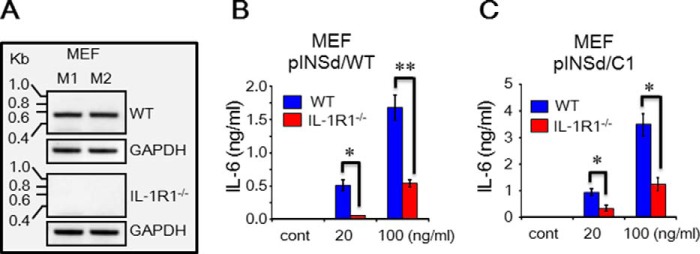
**pINSd/WT and /C1 activity with MEFs of IL-1R1-deficient mice.** *A*, RT-PCR of mouse IL-1R1 confirmed that IL-1R1 expression was absent in the MEFs of IL-1R-deficient mice. *A* and *B*, the MEF cells of WT and IL-1R-deficient mice were treated with pINSd/WT and /C1 for IL-6 production. Data in *B* and *C* are comparisons of WT and IL-1R1-deficient mice. Data are mean ± S.E. (*error bars*). *, *p* < 0.05; **, *p* < 0.01 from duplicates. *cont*, control.

**FIGURE 9.**
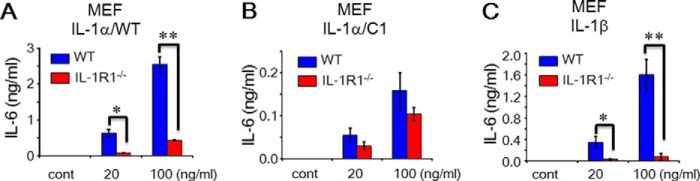
**IL-1α/WT and /C1 activity with MEFs of IL-1R1-deficient mice.** *A–C*, the MEF cells of WT and IL-1R-deficient mice were treated with IL-1α/WT, IL-1α/C1, and IL-1β for IL-6 production. Data in *A–C* are comparisons of WT and IL-1R1-deficient mice. Data are mean ± S.E. (*error bars*). *, *p* < 0.05; **, *p* < 0.01 from duplicates. *cont*, control.

We were interested in investigating pINSd-mediated inflammatory cytokine production using MEFs of IL-1R1-deficient mice. We first examined mouse IL-1R1 mRNA expression in the MEFs of WT and IL-1R1-deficient mice. As shown in Fig. 8*A*, IL-1R1 expression was observed in MEFs of WT mice but not in IL-1R1-deficient mice. Similar to the previous results of human cell assays, the pINSd/C1 was approximately two times more active than pINSd/WT in MEFs of WT (Fig. 8, *B* and *C*). As shown in Fig. 8, *B* and *C*, pINSd/WT- and /C1-mediated mouse IL-6 production was significantly impaired in MEFs of IL-1R1-deficient mice. These data further confirmed that pINSd stimulates IL-1R1 to induce inflammatory cytokines. Finally, the activity of IL-1α/WT, IL-1α/C1, and IL-1β was tested with MEFs of IL-1R1-deficient mice. Similar to the result in Fig. 5, IL-1α/C1 impaired IL-6 production in WT MEFs (Fig. 9*B*) compared with IL-1α/WT (Fig. 9*A*). The pattern of IL-1α/WT, IL-1α/C1, and IL-1β activity was similar to that of pINSd (Fig. 8) in the MEFs of IL-1R-deficient mice (Fig. 9).

## Discussion

To date, INS studies have focused only on regulating glucose levels despite the diverse evidence suggesting that the immunological functions of INS are also important for diabetes (26, 29–31). Here we expressed recombinant pINS protein to investigate its activity. The results of immunological pINSd activities were reproducible with various cell types, and it was found that only pINSd possesses the immunological capability to induce inflammatory cytokines with comINS and pINSm unable to induce them. These data provide direct evidence for the first time that pINS induces an immune response through the induction of inflammatory cytokines.

A recent study has provided a rapid test of therapies designed to preserve pancreatic β-cells in type 1 diabetics, but it is hampered due to limited availability of sensitive β-cell health biomarkers (32). In this study, the levels of the proinsulin:C-petide ratio and HSP90 were increased in type I diabetes patients, and HSP90 levels were also increased 4-fold in islets isolated from non-obese prediabetic mice compared with matched CD1 controls. This study suggested that β-cell stress can be measured using proinsulin:C-petide ratios and HSP90 to reduce β-cell stress in new onset type I diabetics. Further studies with diabetic patients are necessary to evaluate the immunological functions of pINSd in type 1 and type 2 diabetic patients.

Human mature comINS contains 51 amino acids with A and B chains linked by disulfide bonds, whereas pro-INS contains 86 amino acids before cleavage of the C-peptide between the B and A chains. Prepro-INS contains a hydrophobic 24-residue signal peptide that directs the nascent polypeptide chain to the rough endoplasmic reticulum. The signal peptide is cleaved as the polypeptide is translocated into the lumen of the rough endoplasmic reticulum, forming pINS (33). The amino acid sequence of INS was first characterized (34), and then its structure was determined by x-ray crystallography (35). The mAb anti-IL-1α recognizing pINSd as well as the overlap in activity between pINSd and IL-1α led us to investigate the sequence homology between pINSd and IL-1α. A careful analysis of the pINS sequence revealed an INS/IL-1α homology motif in the C-peptide. Deletion of the motif changed the biological activities of pINSd and IL-1α, suggesting a critical role of the motif in immunological functions (Figs. 5 and 6). However, further study is necessary to validate the enhancement of pINSd/C1 but not IL-1α/C1, which lost its activity.

The existence of the INS/IL-1α motif in pINSd and IL-1α and its absence in IL-1β could account for the unique activity of IL-1α in cellular metabolism associated with cell growth and cancer that diverges from IL-1β activity, which is limited to inflammatory functions (36, 37). The contradictory reports of the effects of IL-1Ra on IL-1β activity in type 1 and 2 diabetes (38, 39) could be explained by the unique activity of pINSd and IL-1α. Using the precise sequence information of INS/IL-1α motif, development of a neutralizing antibody against the ligands and receptors will shed light on the specific role of pINSd and IL-1α in diabetes.

IL-1Ra completely abolished IL-1β-induced cytokines in all cells types, whereas in contrast the inhibitory effect of IL-1Ra on pINSd- and IL-1α-mediated cytokines was dependent on cell type. We also used IL-1R1-Fc (not shown) to modulate the activity of pINSd in an *in vitro* assay, but these Fc receptors were not sufficient to regulate pINSd activity due to the heterodimeric composition of cytokine receptor on cell membranes (9, 10). Further studies are necessary to evaluate the role of IL-1R2, IL-1R3, and INS receptor in the immunological activity of pINSd, although we have characterized IL-1R1 as a crucial component in pINSd-induced immunological activity.

Metabolic regulation of cell proliferation and immune responses against pathogens is requisite for survival of the host. Our discovery of the immunological activity of pINSd suggests for the first time a direct connection between metabolic regulation and the immune response.

## Experimental Procedures

### 

#### 

##### Construction of Plasmid Vector

We obtained human INS cDNA from Dharmacon (Lafayette, CO) and transferred the open reading frame of pINS without the hydrophobic signal peptide of 24 amino acid residues and possessing a His tag at the N terminus into pProEx/HTa (Life Technologies). Human precursor IL-1α, IL-1β, and IL-1Ra cDNAs were cloned as described (40). Mouse IL-1α and IL-1β cDNAs were isolated from LPS-induced Raw 264.7 cells (not shown) and then transferred to an expression vector. The cDNA of IL-1R1 was cloned from A549 cells and then transferred into mammalian expression vector pCAGGs/Neo^4^ for Wish cell stable clones. The sequences of the constructed vectors were confirmed by CosmoGen (Seoul, Korea).

##### Mutagenesis

The cDNAs of the plasmid vector from WT pINS and mature IL-1α were used as templates to generate mutant pINS/C1 as well as IL-1α/C1. We designed specific sense and reverse primers that overlapped with the deletion site and removed the C1 sequence (INS/IL-1α) motif (Fig. 3*D*) as described (40). The sequences of mutant vectors were confirmed (CosmoGen).

##### Expression of Recombinant Protein

pINS/WT and /C1 as well as mature IL-1α/WT and /C1 were expressed in *E. coli*. We purified the recombinant proteins from the inclusion bodies by lysing with a phosphate buffer for 3 h. The supernatant of the recombinant protein was passed through a mini-Talon column and eluted with imidazole (0.3 m). The eluted fractions were directly applied to HPLC. The peaks of each fraction (*A*_280 nm_) were visualized with silver staining (not shown). Human IL-1Ra, mouse IL-1α, and IL-1β were expressed as described (41). Similarly sized bands were pooled and lyophilized for further testing of purity and quantity evaluation. The comINS was obtained from Green Cross (Kyeonggi-do, Korea).

##### Cells and Assays

A549, Huvec, Wish, CHO, and THP-1 cell lines were obtained from American Type Culture Collection (ATCC) and maintained according to the instructions provided. Primary human WBCs (1:4 dilution with RPMI 1640 medium), PBMCs, and PMN leukocytes (5 × 10^5^/well in a 96-well plate) were isolated and treated with various stimuli with concentrations as defined in each figure. For MEF cell isolation, 13.5–14.5-day embryos were minced with forceps for single cell suspension. The isolated MEF cells were cultured in DMEM. Human PBMCs were isolated by density centrifugation of blood over Ficoll-Paque^TM^ PLUS (GE Healthcare). PBMCs were washed twice with saline (0.9% sodium chloride) and resuspended in a culture medium (RPMI 1640 medium). The cell culture supernatant was harvested at different times for cytokine assay. Human cytokines (IL-6, IL-1α, IL-1β, and TNFα) and mouse IL-6 were measured with ELISA kits from R&D Systems according to the manufacturer's instructions. The data are expressed as means ± S.E. Statistical significance of differences was analyzed by unpaired, two-tailed Student's *t* test. Values of *p* <0.05 were considered statistically significant. All data shown are representative of at least five independent experiments.

##### Cell Line for Overexpression of IL-1R1

Prior to transfection of pCAGGs/Neo-human IL-1R1 into parent Wish cells, we examined the expression of IL-1R1 with RT-PCR, Western blotting, and FACS analysis (not shown). Wish cells were transfected with empty pCAGGs or pCAGGs/Neo-human IL-1R1 plasmid DNA (2 μg) using Lipofectamine® 2000 from Life Technologies. A positive clone of Wish IL-1R1 was first screened with RT-PCR and then further confirmed with Western blotting and FACS analysis (not shown).

##### Western and Dot Blotting

Wish IL-1R1/C-3 and mock control cells were cultured and then harvested for Western blotting. Cell lysate was obtained by directly adding lysis buffer (Cell Signaling Technology, Danvers, MA) to the plate after removing the cell culture medium with PBS washing. For the A549 cell supernatant of the pINSd-treated cells, standalone pINSd (100 ng/ml) or pINSd preincubated with trypsin (20 ng/ml; Sigma-Aldrich) was used at the indicated times without FBS. The cell lysate (100 μg) or the supernatant (60 μl) was mixed with loading buffer, boiled for 10 min, and loaded for 10% SDS-PAGE. Proteins were separated by electrophoresis and blotted onto nitrocellulose membrane (Whatman). For dot blotting, recombinant proteins were dropped on a nitrocellulose membrane, and the amount was defined on each blot. The primary antibodies (1 μg/ml rabbit polyclonal anti-IL-1α and mAb anti-IL-1α) were incubated at 4 °C overnight. After washing, the membranes were incubated in horseradish peroxidase (HRP)-coupled respected anti-IgG (Jackson ImmunoResearch Laboratories) and then Supex (Neuronex, Seoul Korea) and an LAS-4000 imaging device (Fujifilm, Japan) were used to develop the blot.

##### Monoclonal and Polyclonal Antibody Development

Mice were maintained in microisolator cages in a temperature- and humidity-controlled barrier facility and provided with autoclaved water and food. All experiments were approved by the Institutional Animal Care and Use Committee at Konkuk University. We developed monoclonal antibodies against IL-1α protein. The anti-IL-1α antibody-producing B cells were generated by immunizing BALB/c mice four times with 50 μl of recombinant protein (10 μg) and an equal volume of adjuvant (Gerbu, Gaiberg, Germany). The titration of immunized mouse sera was confirmed by using direct ELISA with the antigen. The splenocytes of immunized mice were fused with mouse myeloma cells. The immunoglobulin isotypes of the selected mAb clones were determined using an Immuno-Type^TM^ mouse mAb isotyping kit (BD Biosciences) according to the manufacturer's instructions. The mAbs were purified using protein G-agarose (Kirkegaard & Perry Laboratories, Inc., Gaithersburg, MD), and their purity was confirmed using Coomassie Blue-stained SDS-polyacrylamide gels (not shown). The mAbs were aliquot and stored at −80 °C until use.

##### IL-1α ELISA Kit

We developed an IL-1α sandwich ELISA by using the anti-IL-1α mAb (1 μg/ml) as a capture antibody and affinity-purified rabbit anti-IL-1α polyclonal antibody (0.1 μg/ml) as a detection antibody. After washing, the wells were incubated with horseradish peroxidase-conjugated goat anti-rabbit IgG (Jackson ImmunoResearch Laboratories) for 1 h and then developed with 3,3′,5,5′-tetramethylbenzidine liquid substrate (Sigma-Aldrich). The absorbance at 450 nm was measured using an ELISA plate reader (Molecular Devices, Sunnyvale, CA).

## Author Contributions

H. J., S. L., and E. K. designed the study, analyzed the data, and performed the experiments. Jo. L., S. Y., A. K., Ju. L., S. J., Somi K., M. K., H. K., and D.-K. C. performed the experiments. J. H., S. B., B. K., Y. L., and Y.-S. K. analyzed the data. Soohyun Kim designed the study, supervised the project, and wrote the manuscript.

## Footnotes

4 S. Lee, E. Kim, H. Jhun, J. Hong, A. Kwak, S. Jo, S. Bae, J. Lee, B. Kim, J. Lee, S. Youn, S. Kim, M. Kim, H. Kim, Y. Lee, D.-K. Choi, Y.-S. Kim, and S. Kim, unpublished data.

## Footnotes

3 The abbreviations used are:

INS: insulin
pINS: pro-INS
pINSd: proinsulin dimer
IL-1R: IL-1 receptor
IL-1Ra: IL-1 receptor antagonist
PBMC: peripheral blood mononuclear cell
pINSm: proinsulin monomer
Huvec: human umbilical vein endothelial cell
comINS: commercial INS
PMN: polymorphonuclear
MEF: mouse embryo fibroblast
WBC: whole blood cell
C: clone.
